# Excess mortality during COVID-19 pandemic in Bangladesh – evidence from a rural survey

**DOI:** 10.7189/jogh.14.05031

**Published:** 2024-10-25

**Authors:** Aniqa Tasnim Hossain, Ema Akter, Abu Bakkar Siddique, Md Hafizur Rahman, Shafiqul Ameen, Sabrina Jabeen, Ridwana Maher Manna, Md Alamgir Hossain, Qazi Sadeq-ur Rahman, Anisuddin Ahmed, Shabnam Mostari, Anir Chowdhury, Syed Moshfiqur Rahman, Mohammod Jobayer Chisti, Daniel Cobos, Shams El Arifeen, Ahmed Ehsanur Rahman

**Affiliations:** 1 Maternal and Child Health Division, International Centre for Diarrhoeal Disease Research, Dhaka, Bangladesh; 2 Department of Women's and Children's Health, Uppsala University, Uppsala, Sweden,; 3 Aspire to Innovate (a2i) Programme, Dhaka, Bangladesh; 4 Nutrition and Clinical Services Division, International Centre for Diarrhoeal Disease Research, Dhaka, Bangladesh; 5 Health System and Policy, Swiss Tropical and Public Health Institute, Allschwil, Switzerland

## Abstract

**Background:**

The coronavirus disease 2019 (COVID-19) had a profound impact worldwide. In Bangladesh, the official number of deaths for COVID-19 was around 29 000. However, many countries including Bangladesh experienced substantial underreporting of COVID-19 deaths due to lack of complete national civil registration system. This study aims to estimate excess mortality in 2020, identify risk factors, and determine leading causes of death in Bangladesh.

**Methods:**

In February 2021, we conducted a cross-sectional household survey in Sitakunda, a subdistrict of Chattogram, identifying deaths from January 2018 to December 2020. Excess mortality was quantified using the p-score and incidence rate ratio (IRR) utilising Poisson segmented regression. We employed the InterVA-5 algorithm to attribute causes of death. Proportional distribution and cause-specific mortality rates (CSMR) per 100 000 individuals were compared between pre-pandemic and pandemic periods.

**Results:**

Among 1748 deaths from 25 669 households, we found 1.4 (95% confidence interval (CI) = 1.2–1.4) times excess mortality in 2020 compared to 2018–2019. Leading causes of death in 2020 included cardiac disease (CSMR = 121.0, CI = 115.8–127.3), stroke (CSMR = 108.0, CI = 102.6–114.0), and acute respiratory infection (CSMR = 61.0, CI = 55.1–66.5), all displaying significantly higher mortality rates than in previous years. Older age (IRR = 1.6), less education (IRR = 1.8), and lower socio-economic groups (IRR = 2.1) had higher mortality rates in 2020 compared to pre-pandemic years.

**Conclusion:**

Our study suggests high rural excess mortality during COVID-19 including cardiac disease, stroke and acute respiratory infection as the leading causes of deaths. We require targeted strategies to identify high-risk patients with comorbidity and social vulnerabilities that contribute to mortality to guide the preparedness strategy for future pandemics.

The coronavirus disease 2019 (COVID-19) was declared as a public health emergency of international concern in January 2020 by the World Health Organization (WHO). Reliable data and accurate measurements are paramount to understand the magnitude and impact of this pandemic for each region and country to guide policymakers in health systems planning and optimal distribution of resources [1]. Government has established different mechanisms to track deaths and infections due to COVID-19 [2]. In Bangladesh, the officially reported number of COVID-19 deaths was around 29 000. However, in the majority of countries, including those with high-incomes, these numbers are grossly underreported due to the lack of effective routine mortality surveillance systems [3]. Underreporting of deaths has significant implications for public health and policy-making. During the COVID-19 pandemic, underreporting hindered accurate understanding of the virus spread and impact, complicating efforts to control the outbreak and plan for the allocation of vaccine and therapeutics. Even though the reported deaths give some indication of the pattern of mortality it only tells a tip of the iceberg of the true excess mortality. Excess mortality is defined by both the direct and indirect effects of the pandemic. It is the degree to which currently measured mortality exceeds baseline levels. In the context of COVID-19, increases in total mortality are attributed to the overall effects of the pandemic [4].

The gaps between reported deaths and actual deaths attributable to the disease exist for multiple reasons [5]. During the early phase of pandemic, testing capacity was low in Bangladesh and the system needed widespread medical certification of cause of death [6]. Later, this situation gradually improved. However, by the time we could ensure adequate testing capacities, we missed many of the COVID-19 deaths to be reported [7]. Also, many low and middle-income countries (LMIC), including Bangladesh, lack the capacity and efficiency to establish precise and timely estimates of mortality, critical for public health decision making during the pandemic [8]. The civil registration and vital statistics (CRVS) system in Bangladesh is not very strong in terms of quality and completeness [9]. Moreover, the death registration system in Bangladesh is still paper-based [10]. These factors likely contributed to significant delay in the reporting of deaths, which led to delays in appropriate responses by policymakers. Lastly, many different organisations worked on reporting and tracking mortality using stand-alone systems when the pandemic hit [6]. The lack of collaboration between these efforts in Bangladesh was a missed opportunity to get a comprehensive understanding of the true burden [8].

Many individual health-related risk factors for increased risk of death from COVID-19 have been identified, but less is known about characteristics and causes that make communities resilient or vulnerable to the mortality impacts of the pandemic [11]. We also need to understand the changes in causes of deaths before and during COVID-19 to get a holistic understanding of the excess mortality. Verbal autopsy (VA) is used in ascertaining causes of deaths as part of strategies to capture excess mortality [12].

The objective of this study is to produce reliable estimates of excess mortality during COVID-19 through a household survey in a rural subdistrict of Bangladesh comparing with the pre-COVID-19 period. We aim to present the leading causes of death, and assess risk factors associated with mortality, to understand the impact of the COVID-19 pandemic by each of the causes and risk factors in this community.

## METHODS

### Study design and site

**Figure 1:**
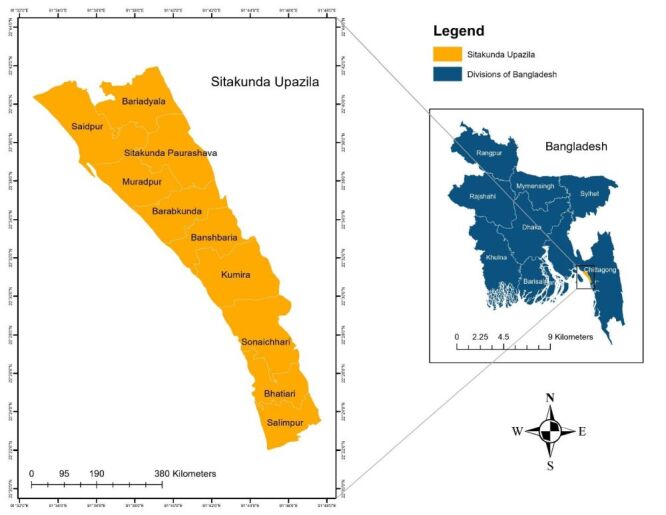
Map and location of the survey site.

We conducted a cross-sectional population-based household survey in February 2021 in Sitakunda (**Figure 1**), a subdistrict of Chattogram in Bangladesh. Sitakunda is a subdistrict of 387 832 population comprising of 483.8 km^2^ area. Among the population of Sitakunda 48% are men and 52% women. The proportion of literate people in this area is 60% [13]. Economic development in Sitakunda is significantly influenced by the roads and the railway. The area is primarily agricultural and it also hosts world's largest ship-breaking industry. Many workers in this industry come from impoverished areas, seeking employment opportunities despite the hazardous working conditions. These workers often face serious health risks, including accidents and exposure to toxic substances. The population of Sitakunda is diverse, with a mix of permanent residents and migrant workers, reflecting the area's economic dynamics.

Sitakunda has several health care facilities, including the Sitakunda Upazila (subdistrict) Health Complex with 50-bed facility. Other facilities include union sub-centres and few private facilities [14]. Despite these resources, the health system struggles with issues such as inadequate infrastructure, limited access to trained health care providers, and the need for better emergency response capabilities.

### Study population

The study population included all individuals who were alive at the end of December 2017 and resided within the study site boundaries. We excluded individuals who permanently migrated out of the study site after December 2017. Data collectors mapped and listed all households within selected clusters of Sitakunda. Clusters were selected using probability proportional to size sampling (PPS) [15]. In the Bangladesh Demographic and Health Survey (BDHS), a household is defined as a person or group of related and unrelated persons who usually live together in the same dwelling unit(s), who have common cooking and eating arrangements, and who acknowledge one adult member as head of the household. A member of the household is any person who usually lives in the household.

### Sample size

Figure S1 in the **Online Supplementary Document** shows the flowchart of included sample in this study. We have selected 60 clusters with approximate size of 300 to 500 households using PPS. Sample size was determined using age-specific death rate for individuals aged 40 years and above to detect 25% relative change from pre-pandemic to pandemic period. We visited 25 669 households with 113 548 population. 25 560 households gave consent. We identified 1748 deaths in 2018, 2019 and 2020 from these households. Among them 1411 deaths were of the people aged 40 years and above.

### Data collection method

The household survey was conducted using structured questionnaires administered by locally recruited trained data collectors between February and June 2021. Data collectors conducted interviews with the household heads following informed consent to collect basic demographic information regarding all regular members of the listed households. In addition, vital status of each member of the household during the study period was recorded during the survey. Verbal autopsy was performed for any reported deaths that occurred during the study period. Data was collected using electronic tablets.

### Data analysis

We used a standard statistical package (Stata version 14, Texus, USA, 2015) for data analysis. The following disaggregating variables were included as baseline characteristics: age, sex, educational status (literate or illiterate) and socioeconomic (SES) status. Three categories of SES (low, middle, and high) were derived using principal component analysis based on ownership of household goods.

We used proportional all-cause excess mortality score (*P*-score) as a measure of excess mortality. *P*-score was calculated as the percentage difference between the number of deaths in 2020 and the average of the number of deaths in 2018 and 2019. The monthly count of deaths from January 2018 to December 2020 (36 time points in three years) was plotted to assess any trends. There are few assumptions for computing *P*-score. The expected number of deaths (baseline) is accurately estimated from historical data. Deaths are consistently reported over time. Any changes in reporting practices or delays in death registration can affect the accuracy of the *P*-score. The time periods used for calculating expected and observed deaths are comparable. The population size and structure remain relatively stable over the period of analysis.

We performed segmented regression to analyse the monthly death count data with Poisson distribution [16]. Segmented regression is used in interrupted time series (ITS) studies when there is data about an outcome over time to understand how and if the outcome changes after the introduction of something new at one specific point in time [17,18]. In our study, we considered an indicator for time occurring before (COVID-19 = 0) or after (COVID-19 = 1) the COVID-19 pandemic. We considered monthly deaths count as the outcome variable. As data were collected over time, we adjusted the autocorrelation and seasonality in the segmented regression. We performed segmented regression for each of the category of the selected background characteristics (age, sex, religion, education, and SES). The incidence rate ratio (IRR) with 95% CIs is used in Poisson regression to measure the effect of a predictor on the incidence rate of the outcome variable. The IRR is the ratio of the incidence rate of the outcome variable for a unit increase in the predictor variable compared to the incidence rate of the outcome variable for the predictor variable at its baseline value.

There are few assumptions of Poisson Segmented Regression. First, the response variable is assumed to follow a Poisson distribution. This means the data should represent counts of events occurring within fixed intervals of time. Second, the occurrences of events are assumed to be independent. Third, the logarithm of the expected value of the response variable can be modelled as a linear combination of the predictor variables. Fourthly, the mean and variance of the response variable are assumed to be equal. The relationship between the predictor variables and the response variable can change at certain points, known as breakpoints.

We have also conducted Cox-Proportional Hazards Model to assess hazards ratios for COVID-19 adjusted for other covariates such as sex, religion, education, age and socioeconomic status.

We conducted a tablet-based Verbal Autopsy using the WHO standard Verbal Autopsy (2016) tool version 1.5.3, updated for the context of COVID-19 pandemic in 2020 [19,20]. The additional questions related to COVID-19 which were added in the generic WHO-VA tool can be found at the following link: https://www.who.int/healthinfo/statistics/verbalautopsystandards/en/. Causes of death was assigned to each death using the InterVA-5 model [21,22]. The InterVA-5 algorithm is considered appropriate for attributing causes of death for several reasons. It is developed to align with the WHO-2016 verbal autopsy standard. Evaluations have shown high concordance between causes of death assigned by InterVA-5 and those determined in tertiary hospitals, indicating its reliability [21]. InterVA-5 is designed to handle large quantities of verbal autopsy interviews efficiently, making it suitable for use in regions with high mortality rates.

Causes of deaths were reported using descriptive statistics includes cause-specific mortality rate (CSMR) and proportion with 95% CIs.

## RESULTS

**Table 1** presents the background characteristics of the alive and dead members of the selected households in our sample. Among 113 541 alive members three-fourth were educated, one-fourth aged 40 years and above. Among members who died between 2018 and 2020, 59% did not have education and 81% aged 40 years and above.

**Table 1:** Background characteristics of the identified alive and dead members in the sampled households*

Characteristics	Alive		Death	
	**n**	**%**	**n**	**%**
**Sex**				
Female	56 610	49.9	714	40.9
Male	56 931	50.1	1034	59.2
**Religion**				
Islam	96 500	85.0	1487	85.1
Others religion	17 041	15.0	261	14.9
**Education**				
No education	28 307	24.9	1038	59.4
Educated	85 234	75.1	710	40.6
**Age**				
0 to 5 y	12 884	11.4	122	7.0
6 to 19 y	30 540	26.9	70	4.0
20 to 39 y	41 060	36.2	145	8.3
40 y and above	29 057	25.6	1411	80.7
**Wealth status**				
Low	45 648	40.2	674	38.6
Middle	36 034	31.7	535	30.6
High	31 859	28.1	539	30.8

*Alive, n = 113 541; dead, n = 1748.

### All cause excess mortality during COVID-19

In 2018, 493 deaths were identified in Sitakunda of which 408 were adults aged 40 years and above (Figure S2 in the **Online Supplementary Document**). In 2019 we identified 494 deaths, of which 388 were adults 40 years and above. In 2020 the number of deaths for all age groups was 761, including 615 adults ≥40 years. Overall, we observed 54% excess mortality in 2020 compared to the yearly average for 2018 and 2019.

**Figure 2:**
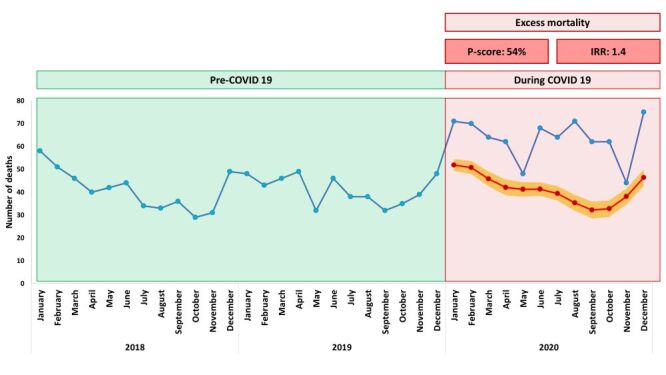
Monthly death 2018–2020 (expected for 2020 using fitted regression for 2018 and 2019) for all ages.

**Figure 3:**
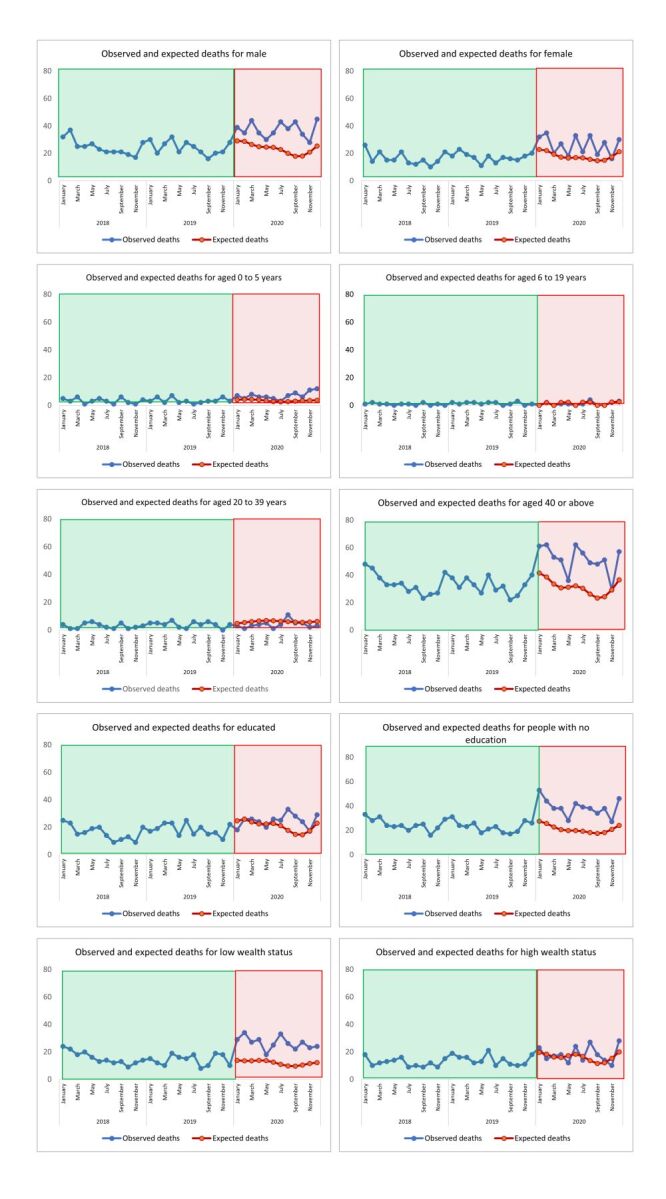
Observed and expected monthly deaths using Poisson segmented regression for interrupted time series analysis stratified by different groups.

**Figure 2** and **Figure 3** present the observed deaths for 2018 and 2019, and both the observed and expected monthly deaths with 95% CIs for 2020. Figure S3 in the **Online Supplementary Document** shows the structural break point in the time series that occurred in January 2020. After an initial spike in deaths in early 2020, we observed a decline in deaths starting in May 2020 in Sitakunda. However, the observed monthly deaths remained well above the upper interval of the expected monthly deaths, indicating significant excess mortality in year 2020. We estimated 232 excess deaths per 100 000 people in 2020 based on the previous two years of monthly death count data (2018–2019). The number of deaths that actually occurred each month in 2020 continued to be much higher than the upper limit of the range of deaths that were expected in those months, indicating that there will be a major increase in mortality in the year 2020 for the groups male, female, people aged 40 years and above, people with no education and low wealth status (**Figure 3**). Table S1 in the **Online Supplementary Document** presents the quarterly death counts of years 2018–2020 and calculated *P*-scores for years 2019–2020.

### Mortality incidence rate by baseline group from segmented regression analysis

**Figure 4:**
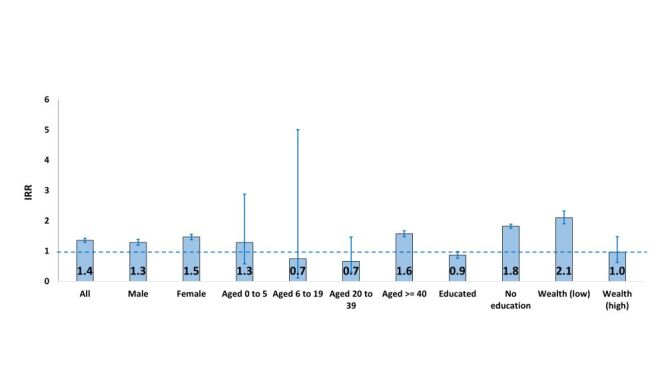
Incidence rate ratios with 95% confidence intervals stratified by the following: male and female, age 0–5, 6–19, 20–39 and ≥40 years, educated and uneducated, and lowest wealth status and highest wealth status determined using Poisson segmented regression for interrupted time series.

**Figure 4** presents the incidence rate ratios (IRR) with 95% CIs for 2020 compared to 2018–2019 for the following strata: male and female, age zero to five years, six to 19 years, 19 to 40 years and 40 years and above, educated and uneducated, and lowest wealth status and highest wealth status determined using Poisson segmented regression for interrupted time series. The overall IRR for death was 1.4 (95% CI = 1.3–1.4) times higher during COVID-19 in year 2020 (**Figure 4**). We observed higher IRRs for death among both males (1.3, 95% CI = 1.2–1.4) and females (1.5, 95% CI = 1.4–1.6). IRR for death was significantly higher among people aged 40 years and above (1.6, 95% CI = 1.5–1.7), people with no education (1.8, 95% CI = 1.7–1.9) and those in the lowest wealth status (2.1, 95% CI = 1.9–2.3). The hazards for COVID-19 adjusted for other covariates using CoxPH model was 1.5 (95% CI = 1.4–1.7) times higher than the pre-COVID-19 period (Table S2 in the **Online Supplementary Document**).

**Figure 5:**
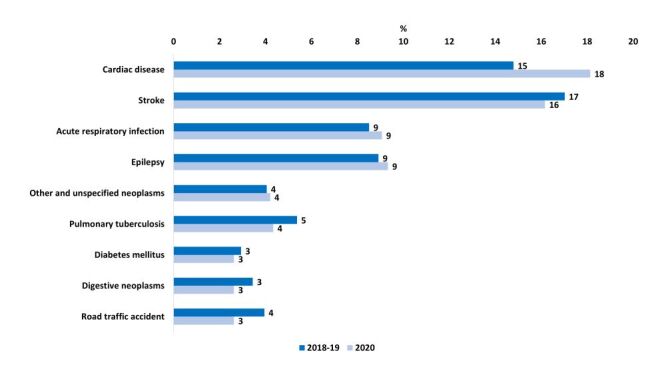
Comparison of major causes of deaths pre- and during COVID-19 period; presented in percent distribution (2018–19, n = 987; 2020, n = 761).

In terms of proportional distributions, we did not observe any major shift in the top causes of deaths between pre and during COVID-19 (**Figure 5**). The conditions that contributed to the majority of the deaths included cardiac disease, stroke, and acute respiratory infection. We identified epilepsy as the fourth major cause of death in Sitakunda. Smaller proportions of multiple other causes contributed to the remainder of identified deaths. (**Figure 5**, Table S3 in the **Online Supplementary Document**).

We observed increased mortality rates for cardiac disease (64.3 to 121.5 per 100 000 population pre-pandemic to pandemic period), stroke (74.0 to 108.3 per 100 000 population), acute respiratory infection (37.0 to 60.8 per 100 000 population) and epilepsy (38.8 to 62.5 per 100 000 population (**Table 2**). Cause-specific mortality rate remained similar for road traffic accidents between the pre-pandemic period (17.2, 95% CI = 12.8–21.6) and during COVID-19 (17.6, 95% CI = 11.9–23.3). Although the leading causes of deaths remained same over the period, the mortality rates were similar in 2018 and 2019 and much higher in 2020 in our analysis (Table S5 in the **Online Supplementary Document**).

**Table 2:** Cause specific mortality rate (per 100 000 population) for all deaths identified in Sitakunda*

Causes	CSMR 2018–19 with 95% CI	CSMR 2020 with 95% CI	Difference between CSMR (2018–19 and 2020)	Percent differences in CSMR (2018–19 and 2020)	*P*-value
Cardiac disease	64.3 (59.9–68.7)	121.5 (115.8–127.3)	57.2	89.0	0.000
Stroke	74.0 (69.6–78.4)	108.3 (102.6–114)	34.3	46.4	0.012
Acute respiratory infection	37.0 (32.6–41.4)	60.8 (55.1–66.5)	23.8	64.3	0.020
Epilepsy	38.8 (34.3–43.2)	62.5 (56.8–68.2)	23.8	61.1	0.023
Other and unspecified neoplasms	17.6 (13.2–22.0)	28.2 (22.5–33.9)	10.6	60.2	0.136
Pulmonary tuberculosis	23.3 (18.9–27.7)	29.1 (23.3–34.8)	5.7	24.9	0.449
Diabetes mellitus	12.8 (8.4–17.2)	17.6 (11.9–23.3)	4.8	37.5	0.411
Digestive neoplasms	15 (10.6–19.4)	17.6 (11.9–23.3)	2.6	17.3	0.669
Road traffic accident	17.2 (12.8–21.6)	17.6 (11.9–23.3)	0.4	2.3	0.949

CI – confidence interval, CSMR – cause-specific mortality rates

*P-value from two rate test of difference between CSMR 2018–19 and CSMR 2020.

## DISCUSSION

Understanding the true impact of the COVID-19 pandemic on mortality is vital for health policies and decision making. Mortality in Sitakunda, Bangladesh increased substantially but heterogeneously during the COVID-19 pandemic. We estimate that 232 excess deaths per 100 000 people occurred in the year 2020 during the COVID 19 pandemic in Sitakunda, representing a 54% increase over the baseline mortality rate. We found a 40% increase in cause-specific risk of death for all causes of death taken together after adjusting for seasonality and autocorrelation. Most excess deaths occurred during the third quarter of the year, when mortality was 87% higher than pre-pandemic observations for the corresponding period. Bangladesh government declared ‘lockdown’ throughout the nation from 23 March to 30 May in 2020 to prevent the spread of infection nationwide. We observed a decline in mortality in our data during this period. Although the overall number of deaths was higher among males, we observed higher excess mortality among females also during COVID-19 compared to the pre-pandemic period. We also observed higher mortality among people with no education and those with lower SES. Cardiac disease, stroke and acute respiratory disease had substantial increases in cause-specific mortality rates during COVID-19 in Sitakunda. These observations provide insight into the impact of the pandemic across demographic groups in a rural setting within the southern region of Bangladesh, where understanding of the true burden associated with COVID-19 was incomplete.

Around 29 000 deaths were officially reported in Bangladesh till May 2023, which is a substantial underreporting of the true mortality impact [22]. Official numbers of COVID-19 cases and deaths in Bangladesh are an underestimate of the real scenario because they are limited to those patients with laboratory confirmed tests and deaths recorded in hospitals [23]. The excess mortality rate for Bangladesh estimated by the Institute for Health Metrics and Evaluation (IHME) was 134.7 per 100 000 population [24], lower than our estimate for Sitakunda. This difference may be due to methodological differences and our focus on a specific region rather than a country-wide approach. Estimates provided by IHME, are based on global models that may use incomplete or less granular data, especially from regions with limited death registration systems. Directly collected local data are more comprehensive and accurate, reflecting the true impact of the pandemic. Differences in how deaths are reported and classified can also contribute to discrepancies. Local authorities have more accurate reporting mechanisms compared to the broader estimates used by IHME. Another survey-based household study in rural Bangladesh found an 8% decrease in mortality in 2020 compared to 2019, suggesting that some rural areas may have been less affected by the pandemic in 2020 compared to more densely populated areas [25]. Another regional all-cause mortality analysis during the COVID-19 pandemic in Chennai in India reported 518 excess deaths per 100 000 people through an observational study, which shows much higher excess deaths in Chennai than what we observed in Sitakunda [26]. Chennai experienced significant strain on its health care system during the pandemic. This could have led to higher mortality rates due to overwhelmed hospitals and delayed treatments for both COVID-19 and other conditions. Moreover, Chennai has diverse socioeconomic conditions, with densely populated areas where the virus could spread more rapidly.

The COVID-19 pandemic has had a significant negative impact on mortality rates in older people living in the rural Bangladesh at Sitakunda. This impact may be attributable to a decreased likelihood of seeking life-saving care or a decreased capacity of the health system to manage non-COVID-19-related health care needs among the older people. Many of these deaths occurred due to chronic diseases like heart disease and stroke, which are more likely to affect older people. The pandemic may have prevented them from traveling the distance to a hospital that could provide them the immediate intensive care services that they needed. Hanifi and colleagues also found 28% increase in excess deaths in older people in 2020 compared to previous five years using data from Matlab Health and Demographic Surveillance [23]. Bangladesh needs to undertake measures to strengthen the CRVS system and national health statistics to monitor morbidity and mortality, especially in an epidemic or pandemic situation to prevent the older adult deaths. Bangladesh should also strengthen its geriatric health services by providing additional resources to make health care services more accessible to its residents irrespective of geographical locations [27].

We also observed SES-related disparities in excess mortality associated with COVID-19 in Sitakunda. During the COVID-19 pandemic in 2020, people with no education and with lower SES had the highest excess all-cause mortality rates. Factors such as occupational risk, socioeconomic factors and housing conditions may have contributed to higher mortality rates in these populations [28,29]. Those living with poorer socio-economic conditions and those with no education may have less opportunities for undertaking measures that reduce transmission such as working from home or limited travel. They may also have higher exposure to infection at work or may be more restricted in terms of accessing health care for COVID-19 and other conditions [30]. Furthermore, they may have lost their work opportunities due to lockdown, hence could not afford the treatment expenses.

There could be multiple reasons for observing higher mortality among older, people with lower socioeconomic group and with no education. Rural areas have fewer health care facilities and professionals compared to urban in Bangladesh. Older and lower socioeconomic groups may face additional barriers, such as transportation issues and financial constraints, limiting their access to timely and adequate medical care. Older individuals and those from lower socioeconomic backgrounds are more likely to have chronic health conditions, which can exacerbate the severity of COVID-19. Lower socioeconomic groups often live in crowded conditions, which can facilitate the spread of the virus and make it difficult to practice social distancing. Also, less educated individuals may have lower health literacy, making it harder for them to understand and follow public health guidelines, recognise symptoms, and seek appropriate care. Another point could be poor nutrition, which is more common in lower socioeconomic groups, can weaken the immune system, making individuals more susceptible to severe illness.

We found excess mortality both in males and females. More deaths occurred among the males in Sitakunda compared to females, though females had higher estimated excess deaths in the COVID-19 period compared to the pre-pandemic period. Many European studies have reported higher COVID-19 related mortality in males compared to females [31]. Sex-differentials in excess mortalities should be examined further to provide knowledge and mechanisms supporting sex equality in public health management and prevention in situations with excess mortality.

Our study also reported the major causes of deaths in Sitakunda during and pre-pandemic period. Although we did not observe any major shifts in the proportional distribution of causes of deaths between these two periods, we found substantial increases in the death rates for cardiac disease, stroke, acute respiratory disease and epilepsy. COVID-19 is likely to have consequences on the cardiovascular health of the people who survive infection [32]. High prevalence of cardiovascular disease is observed among the COVID-19 patients. More than 7% of patients experience myocardial injury from the infection, and for the critically ill patients this proportion is 22% [33]. Therefore, although the cause of death is identified as cardiac disease for many individuals, it is likely that COVID-19 acted as an underlying cause of death for these deceased individuals.

Evidence also suggests positive association between COVID-19 and stroke. A systematic review and meta-analysis conducted in 2020 explored the prevalence of stroke among COVID-19 patients [34]. In a total of 108 571 patients with COVID-19 from 61 studies included in the meta-analysis, 1.4% (95% CI = 1.0–1.9) experienced an acute cerebrovascular event, the most common of which was acute ischemic stroke (87.4%). However, the authors also reported that there was variation in stroke incidence rates among individuals with COVID-19 across the included studies, with higher rates in older patients, those with hypertension, diabetes, coronary artery disease, and in those with severe COVID-19 infection. Asian populations demonstrated the highest stroke rates in this review compared to European and North American populations [35]. The association between stroke and COVID-19 cause is not clearly understood, but sepsis-induced coagulopathy is reported to be associated with COVID-19 which may contribute to endothelial dysfunction, microthrombosis, and stroke [36].

The COVID-19 pandemic has resulted in a large increase of patients with acute respiratory distress in intensive care units worldwide [37]. In our analysis we could not get any individual cause of death assigned as COVID-19 while using interVA algorithm. Acute respiratory disease can be considered a proxy for COVID-19 in this scenario.

Epilepsy is one of the most common chronic neurological conditions [38,39], but we did not expect to see an increase in mortality due to epilepsy during the pandemic compared to previous years, one of the major causes of deaths during COVID-19 period in Sitakunda, Bangladesh. The Center for Disease Control and Prevention (CDC) reported that neurological comorbidities such as epilepsy, could be a risk factor for COVID-19. However, there is a lack of evidence for supporting this association. Some of the researchers tried to assess the association of epilepsy and COVID-19, the evidence is still somewhat scant globally [40]. There is need for further research into COVID-19 and epilepsy to understand this relationship more profoundly. We did not observe any significant change in road traffic accident during and before pandemic. This could be because the survey was conducted in a rural setting where the road vehicle movements may not have shown any changed pattern during pandemic.

### Strengths and limitations

Our study has certain strengths. We have included a large sample of individuals to assess excess mortality during COVID-19 period. Findings from these analyses may be used to make policy decisions focusing on this region to avert deaths in future pandemic. We analysed all causes of death associated with excess mortality in 2020, not just deaths coded to COVID-19, giving a more comprehensive picture of the effects of the pandemic on mortality that is comparable across geographies, as it is not dependent on availability of testing or diagnostic facilities. We have conducted the study in a rural subdistrict with a representative sample. These results are generalisable to the similar contexts in South Asia. This study was undertaken during the COVID-19 period using direct data collection through a cross-sectional survey unlike the global indirect estimates using modelling. Our study has provided important policy implications for initiating routine mortality surveillance by presenting the mortality burden in a crisis periods. Gaining knowledge of this underreported burden, in Bangladesh, we have initiated and assessed feasibility of an innovative mortality surveillance using cemetery records. These initiatives may be taken in other similar settings with low rate of civil registration.

We also acknowledge several limitations of our study. Firstly, we collected data during COVID-19. Remote data collection posed difficulties in reaching diverse populations and ensuring representative samples. Secondly, although, we have undertaken rigorous quality-ensuring mechanisms, we relied on self-reported data for determining number of deaths, which may have introduced some recall error in the data. However, the national health and demographic surveys rely on a recall period of five years to report mortality [41], while our survey collected data on more recent health events (previous three years). The verbal autopsy tool may be limited by self-reported health information but has been validated as an effective tool for cause-specific mortality data collection in settings where medical death certification is unavailable. It may be worth noting that interviewer bias can occur if the interviewer inadvertently influences responses through their questioning style or personal biases. Furthermore, the sensitivity and specificity of VA diagnoses are often limited, particularly for diseases with non-specific symptoms, such as certain cancers or chronic conditions. Thirdly, our sampling strategy adopted the PPS method. PPS sampling gives larger units a higher probability of selection. A potential concern with PPS sampling is that it might not fully capture within-cluster variability if clusters are heterogeneous. However, in our study, the socioeconomic and other key characteristics of the population did not vary significantly across clusters. This relatively low level of variation helped reduce the risk of bias due to cluster heterogeneity. Fourthly, in some of cases household splitting may have caused double reporting of the deaths for the deceased family members in last three years. Double counting of deaths can impact mortality estimates in several ways. It may lead to an overestimation of the number of deaths, which inflates mortality rates. This can distort public health data, mislead policy and resource allocation. However, our data collectors were well trained to check and verify these deaths to avert any double reporting. Fifthly, we have used InterVA-5 algorithm to ascertain causes of deaths [42] which was not specifically tailored to accurately identify COVID-19 as a cause of death. The accuracy of InterVA-5 relies heavily on the quality of verbal autopsy data. Moreover, COVID-19 symptoms and their severity have varied with different variants of the virus. InterVA-5 might not be updated quickly enough to account for these changes. Lastly, we have used *P*-score to present excess mortality [4]. The accuracy of the P-score depends on the baseline period chosen for expected deaths. Changes in population size and structure over time can affect the accuracy of the *P*-score. It may not fully account for seasonal variations in mortality. Comparing *P*-scores across different regions or countries can be challenging due to variations in data quality, health care systems, and reporting practices.

## CONCLUSIONS

We report substantial excess mortality early in the COVID-19 pandemic with disparities across different socio-economic groups in the rural subdistrict, Sitakunda in Bangladesh. These findings have significant implications for future pandemic planning, and highlight the urgency of timely data to address inequities in social determinants of health that increase the risk for death during pandemic in certain populations. Identifying factors that contribute to disparities in mortality, either directly or indirectly attributable to COVID-19, can help decide public health prevention strategies and equitable allocation of resources to progress toward universal health coverage. We recommend investing in improving routine mortality data collection for better estimates. Further research is required to inform long-term strategy to manage different drivers of inequality that may have contributed to mortality during the pandemic.

## Additional material


Online Supplementary Document
