# Supplementary material for: Excess mortality during COVID-19 pandemic in Bangladesh – evidence from a rural survey
Source: J Glob Health. 2024 Oct 25;14:05031. doi: 10.7189/jogh.14.05031 (PMC11503508; doi:10.7189/jogh.14.05031)
Supplement: Online Supplementary Document [file jogh-14-05031-s001.pdf]

Supplementary materials:

Table S1: Quarterly P-score from household survey data at Sitakunda in Bangladesh

Quarters	2018 deaths	2019 deaths	P-score (%) for 2019 compared to 2018	Average of deaths for 2018 & 2019	2020 deaths	P-score for 2020 compared average deaths for 2018 and 2019
q1	155	137	-12	146	205	40
q2	126	127	1	127	178	41
q3	103	108	5	106	197	87
q4	109	122	12	116	181	57
Total	493	494	0	494	761	54

Table S2: Cox-proportional model for hazards ratios of COVID-19 and pre-COVID-19 compared to other covariates

		Unadjusted				Adjusted			
Characteristics	Category	Hazard Ratio	Lower limit	Upper limit	P-value	Hazard Ratio	Lower limit	Upper limit	P-value
Year	2018-2019	Ref				Ref			
	2020	1.50	1.36	1.65	0.000	1.53	1.39	1.69	0.000
Sex	Female	Ref				Ref			
	Male	1.47	1.33	1.62	0.000	1.59	1.44	1.76	0.000
Religion	Others religion	Ref				Ref			
	Islam	1.01	0.88	1.15	0.919	1.06	0.93	1.22	0.389
Education	Educated	Ref				Ref			
	No education	4.56	4.14	5.03	0.000	2.56	2.29	2.86	0.000
Age (in years)	0-5	Ref				Ref			
	6-19	0.21	0.15	0.28	0.000	0.47	0.35	0.65	0.000
	20-39	0.31	0.24	0.40	0.000	0.67	0.51	0.87	0.002

	40 or above	4.09	3.37	4.95	0.000	5.96	4.90	7.24	0.000
Wealth Status	Low	Ref				Ref			
	Middle	1.02	0.90	1.14	0.782	1.13	1.01	1.28	0.032
	High	1.16	1.03	1.30	0.014	1.34	1.19	1.52	0.000

Table S3: Causes of deaths in Sitakunda during and pre-pandemic period, presented in percent distribution and cause-specific mortality rates (CSMR - deaths per 100,000 population)

Causes	Percent distributions 2018-19	Percent distributions 2020	CSMR 2018-19	CSMR 2020
Cardiac disease	14·79	18·13	64·29	121·53
Stroke	17·02	16·16	73·98	108·32
Epilepsy	8·92	9·33	38·75	62·53
Acute respiratory infection	8·51	9·07	36·99	60·77
Pulmonary tuberculosis	5·37	4·34	23·34	29·06
Other and unspecified neoplasms	4·05	4·20	17·61	28·18
Fresh stillbirth	1·93	3·02	8·37	20·26
Diabetes mellitus	2·94	2·63	12·77	17·61
Digestive neoplasms	3·44	2·63	14·97	17·61
Road traffic accident	3·95	2·63	17·17	17·61
HIV/AIDS related death	2·13	2·37	9·25	15·85
Respiratory neoplasms	2·84	2·37	12·33	15·85
Reproductive neoplasms MF	1·62	2·10	7·05	14·09
Diarrhoeal diseases	1·22	1·84	5·28	12·33
Sepsis (non-obstetric)	1·42	1·84	6·16	12·33
Undetermined	1·42	1·45	6·16	9·69
Liver cirrhosis	1·32	1·31	5·72	8·81
Prematurity	1·62	1·31	7·05	8·81
Accid fall	1·11	1·18	4·84	7·93
Birth asphyxia	1·22	1·18	5·28	7·93
Renal failure	1·93	1·18	8·37	7·93
Accid drowning and submersion	1·11	1·05	4·84	7·05
Meningitis and encephalitis	1·52	0·92	6·61	6·16
Other and unspecified infect dis	0·91	0·92	3·96	6·16
Severe malnutrition	1·52	0·92	6·61	6·16
Accid expos to smoke fire & flame	0·41	0·53	1·76	3·52
Acute abdomen	0·00	0·53	0·00	3·52
Neonatal sepsis	0·20	0·53	0·88	3·52
Other and unspecified NCD	0·61	0·53	2·64	3·52
Chronic obstructive pulmonary dis	0·30	0·39	1·32	2·64

**Causes**	**Percent distributions 2018-19**	**Percent distributions 2020**	**CSMR 2018-19**	**CSMR 2020**
Congenital malformation	0·10	0·39	0·44	2·64
Macerated stillbirth	0·20	0·39	0·88	2·64
Abortion-related death	0·41	0·26	1·76	1·76
Assault	0·41	0·26	1·76	1·76
Exposure to force of nature	0·00	0·26	0·00	1·76
Intentional self-harm	0·20	0·26	0·88	1·76
Obstetric haemorrhage	0·61	0·26	2·64	1·76
Oral neoplasms	0·41	0·26	1·76	1·76
Other and unspecified external CoD	0·41	0·26	1·76	1·76
Pregnancy-induced hypertension	0·41	0·26	1·76	1·76
Acid poisoning & noxious subs	0·20	0·13	0·88	0·88
Malaria	0·10	0·13	0·44	0·88
Neonatal pneumonia	0·41	0·13	1·76	0·88
Other transport accident	0·20	0·13	0·88	0·88
Asthma	0·10	0·00	0·44	0·00
Breast neoplasms	0·30	0·00	1·32	0·00
Dengue fever	0·10	0·00	0·44	0·00
Haemorrhagic fever (non-dengue)	0·10	0·00	0·44	0·00
Total	100·00	100·00	434·62	670·20

Table S4: P-value from Dickey-Fuller test for unit root

**Category**	**P-value**
All	0.545
Male	0.356
Female	0.425
Aged 0 to 5	0.971
Aged 6 to 19	0.346
Aged 20 to 39	0.000
Aged >= 40	0.307
Educated	0.287
No education	0.478
Wealth (low)	0.217
Wealth (high)	0.052

Table S5: Cause specific mortality rate (per 100,000 population) for all deaths identified in Sitakunda in year 2018, 2019 and 2020

Causes	Percentage 2018	Percentage 2019	Percentage 2020	CSMR 2018	CSMR 2019	CSMR 2020	P-value (Difference between CSMR 2018 and CSMR 2019)	P-value (Difference between CSMR 2019 and CSMR 2020)
Cardiac disease	14.0	15.6	18.1	60.8	67.8	121.5	0.538	0.000
Stroke	17.8	16.2	16.2	77.5	70.5	108.3	0.511	0.005
Acute respiratory infection	8.7	8.3	9.1	37.9	36.1	60.8	0.816	0.012
Epilepsy	10.5	7.3	9.3	45.8	31.7	62.5	0.115	0.002
Other and unspecified neoplasms	4.5	3.6	4.2	19.4	15.9	28.2	0.613	0.074
Pulmonary tuberculosis	4.5	6.3	4.3	19.4	27.3	29.1	0.241	0.789
Diabetes mellitus	3.0	2.8	2.6	13.2	12.3	17.6	0.842	0.277
Digestive neoplasms	4.7	2.2	2.6	20.3	9.7	17.6	0.074	0.136
Road traffic accident	3.7	4.3	2.6	15.9	18.5	17.6	0.732	0.881

Figure S1: Flow chart of the study sample.

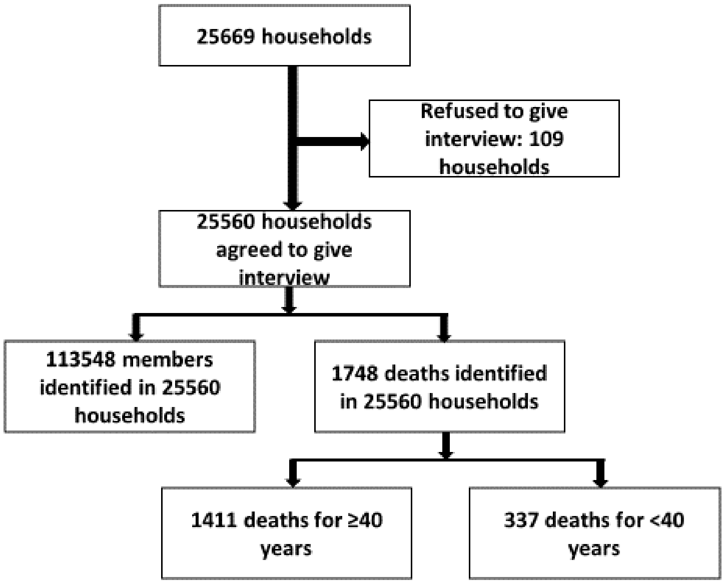

Figure S2: Number of deaths in year 2018, 2019 and 2020 in Sitakunda, Chattogram district, Bangladesh.

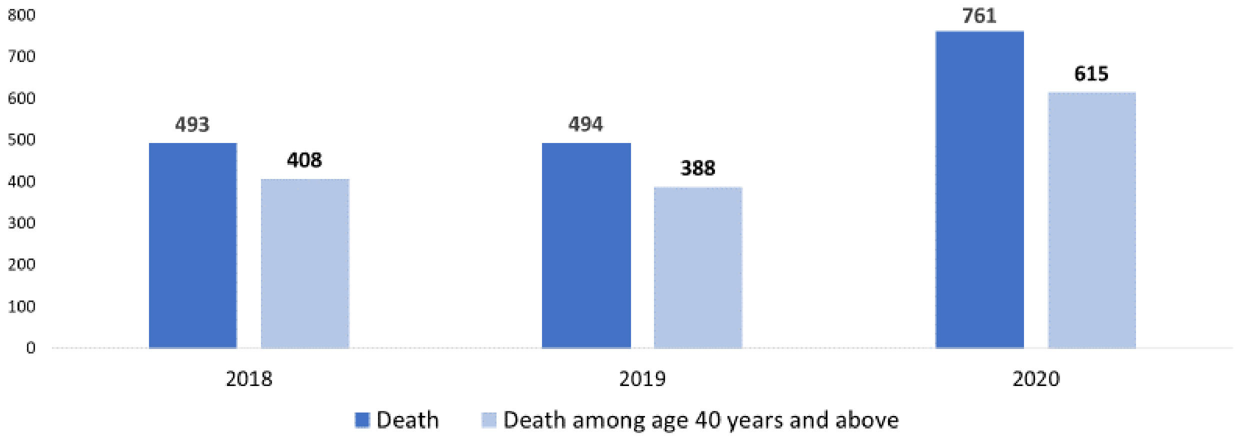

Figure S3: Structural break plot to understand the break point in a time series.

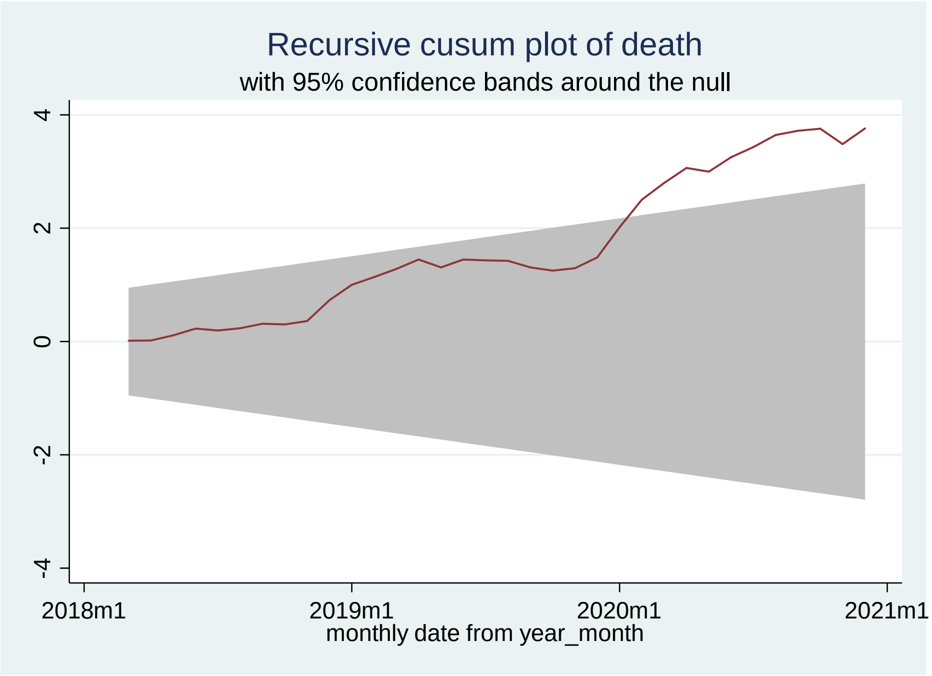

Figure S4: Assumption checking plot to perform segmented regression (All sample: normality checking).

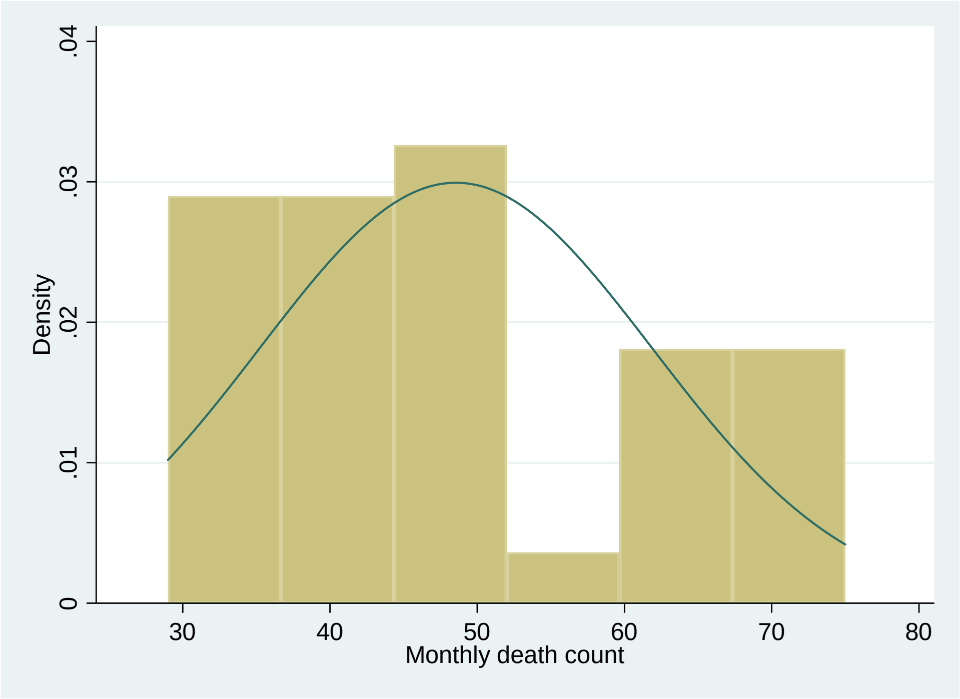

Figure S5: Assumption checking plot to perform segmented regression (All sample: autocorrelation).

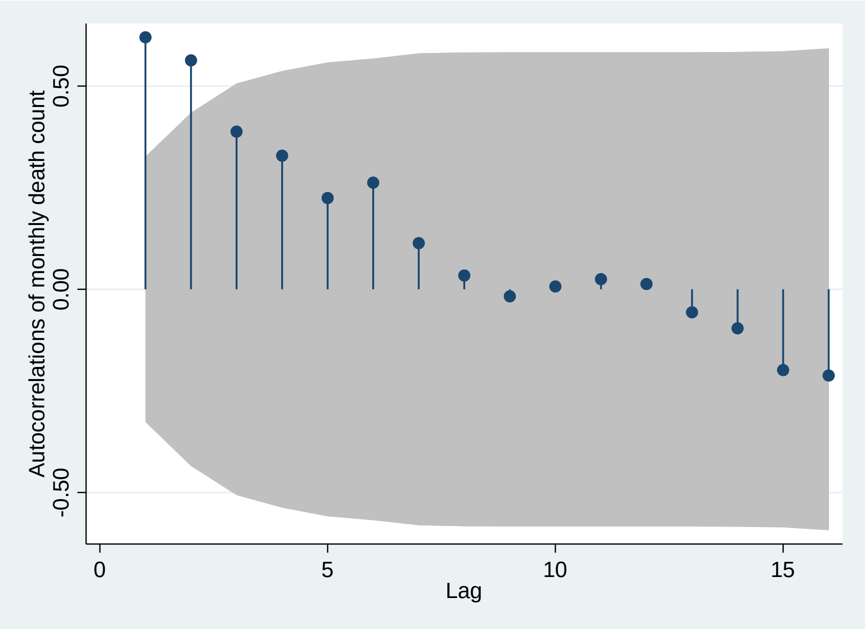

Figure S6: Assumption checking plot to perform segmented regression (Male: normality checking).

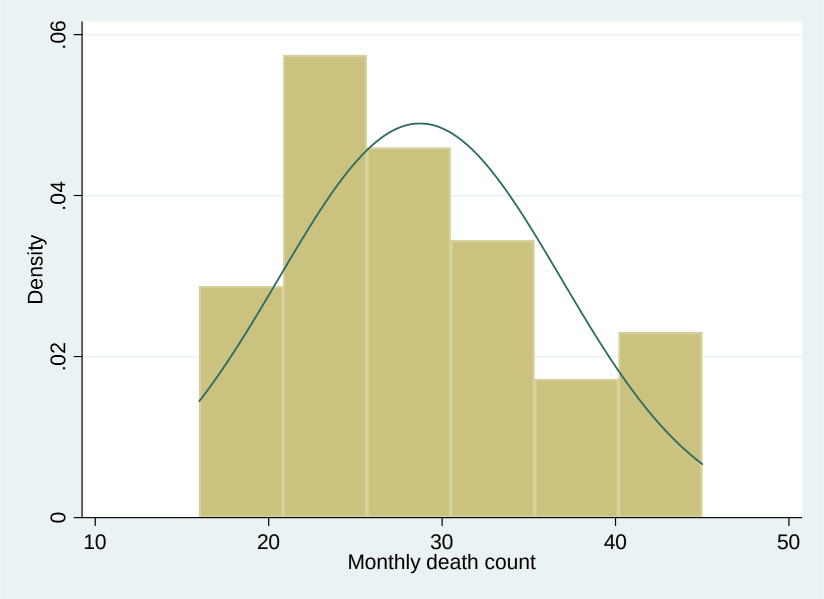

Figure S7: Assumption checking plot to perform segmented regression (Male: autocorrelation).

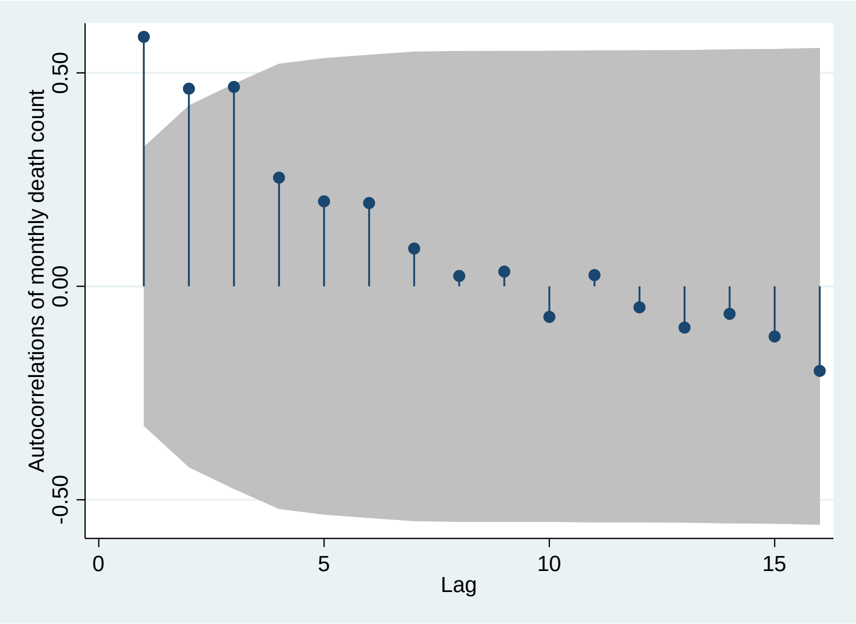

Figure S8: Assumption checking plot to perform segmented regression (Female: normality checking).

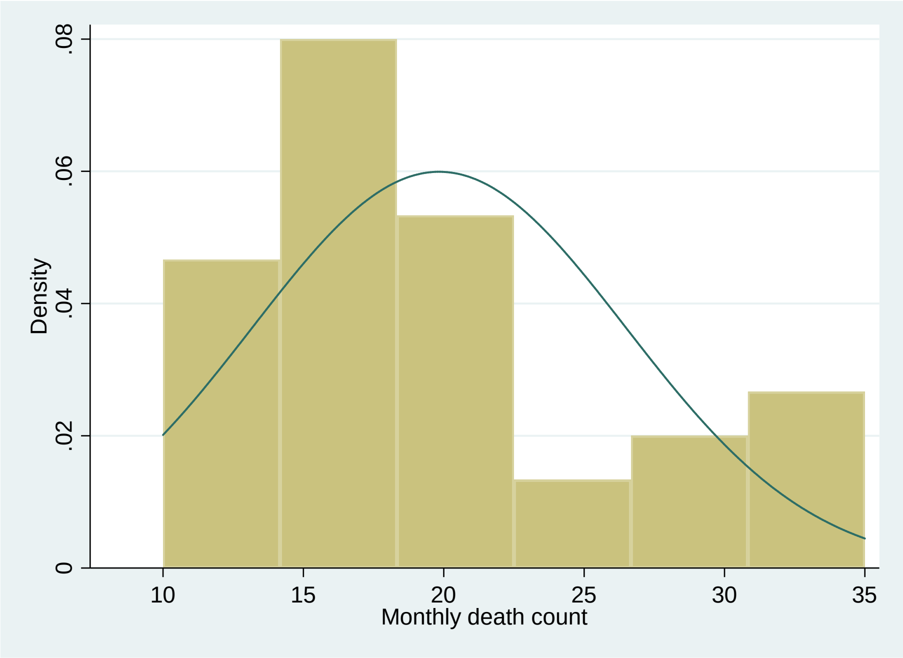

Figure S9: Assumption checking plot to perform segmented regression (Female: autocorrelation).

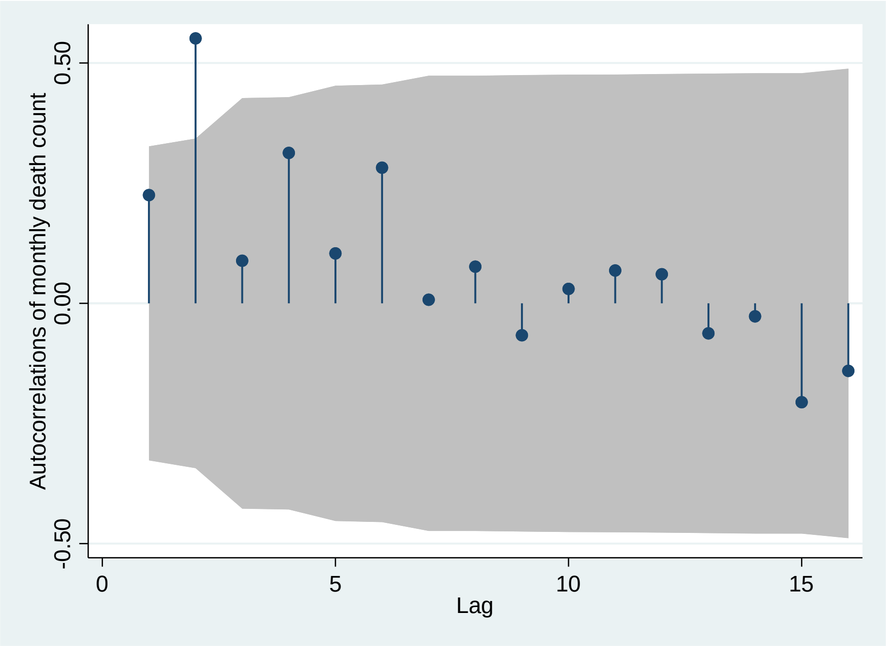

Figure S10: Assumption checking plot to perform segmented regression (Aged 0 to 5: normality checking).

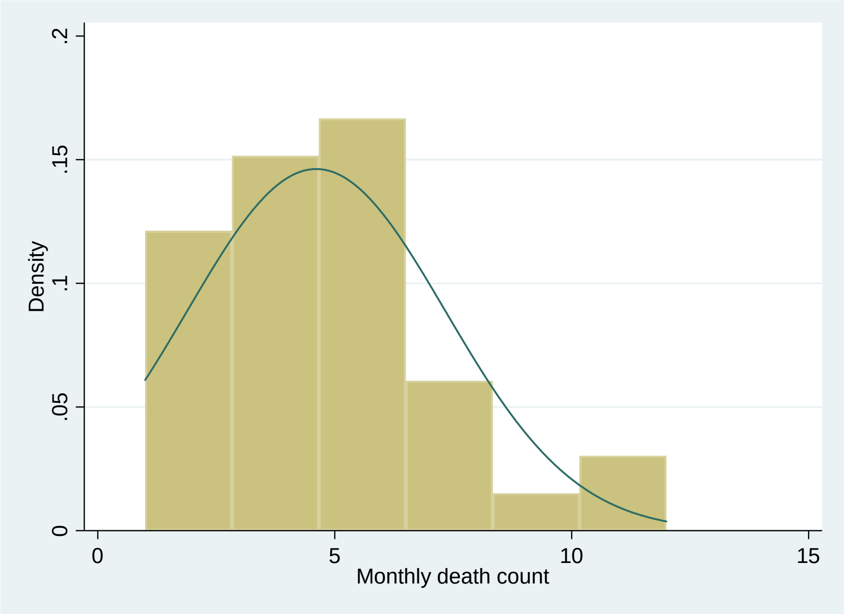

Figure S11: Assumption checking plot to perform segmented regression (Aged 0 to 5: autocorrelation).

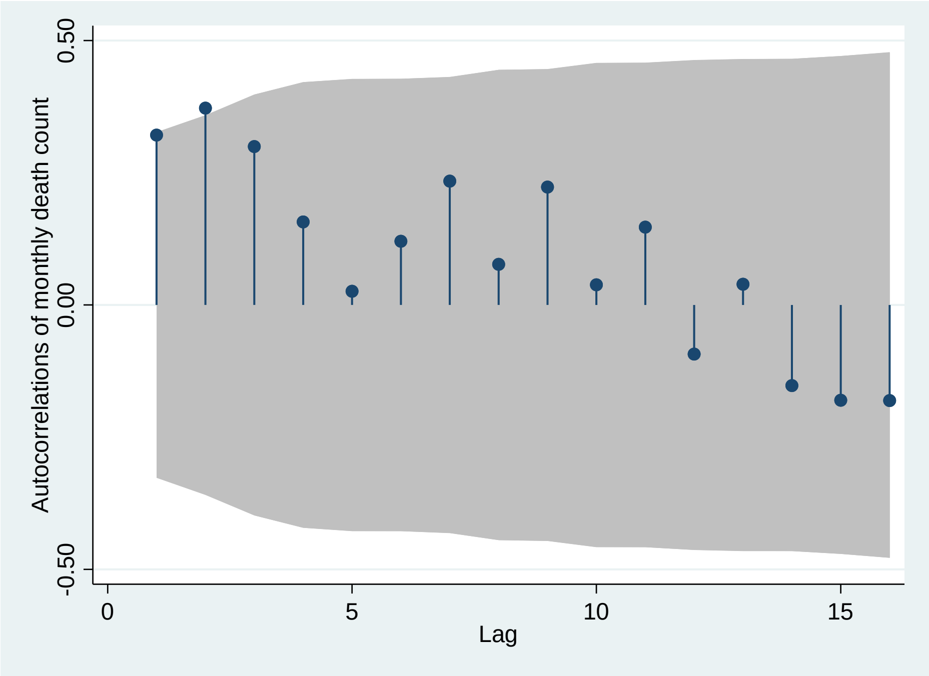

Figure S12: Assumption checking plot to perform segmented regression (Aged 6 to 19: normality checking).

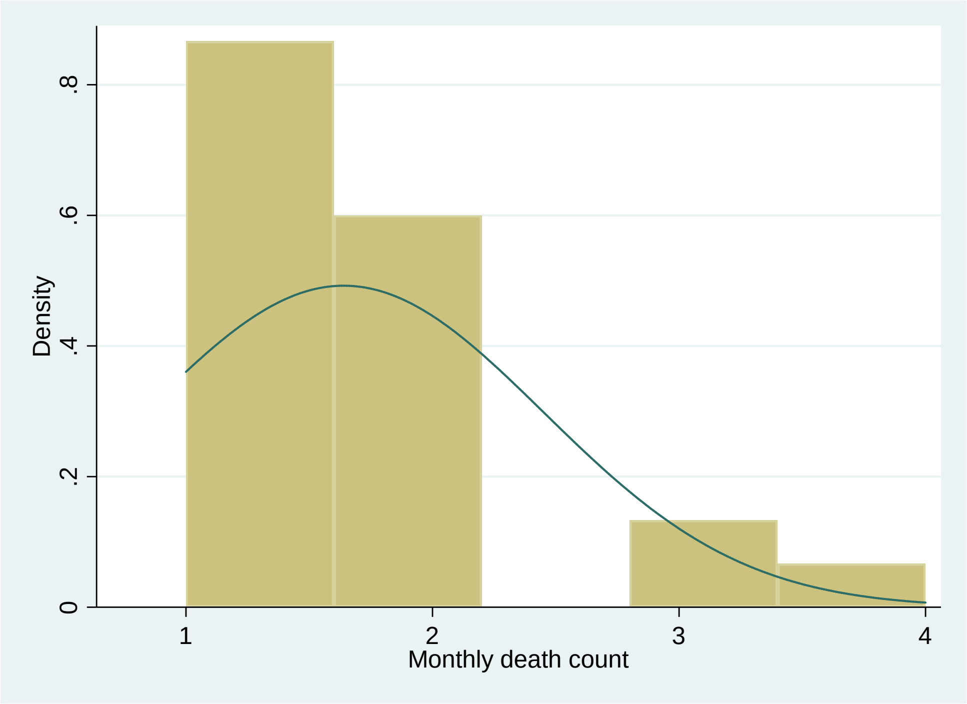

Figure S13: Assumption checking plot to perform segmented regression (Aged 6 to 19: autocorrelation).

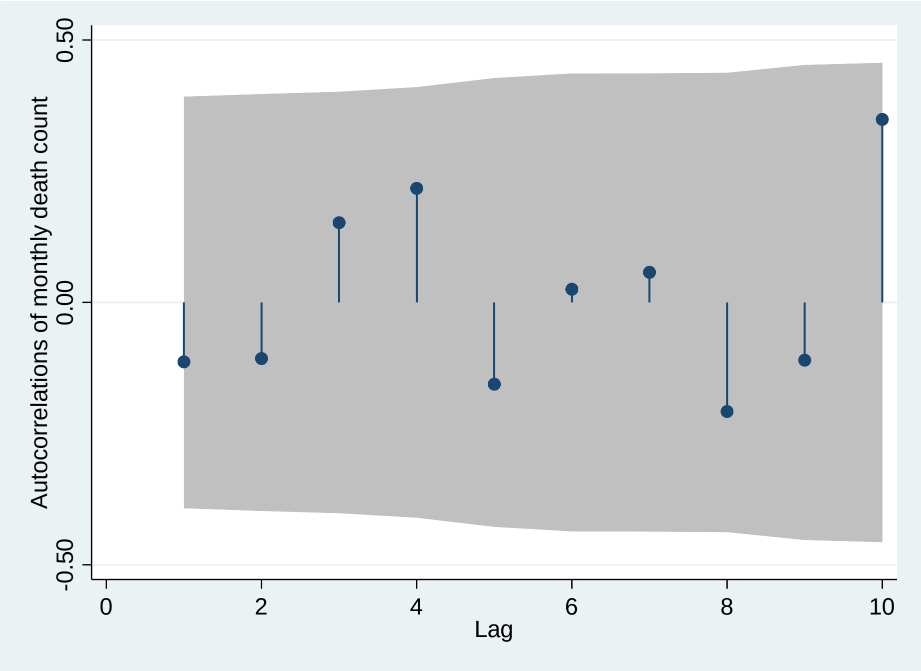

Figure S14: Assumption checking plot to perform segmented regression (Aged 20 to 39: normality checking)

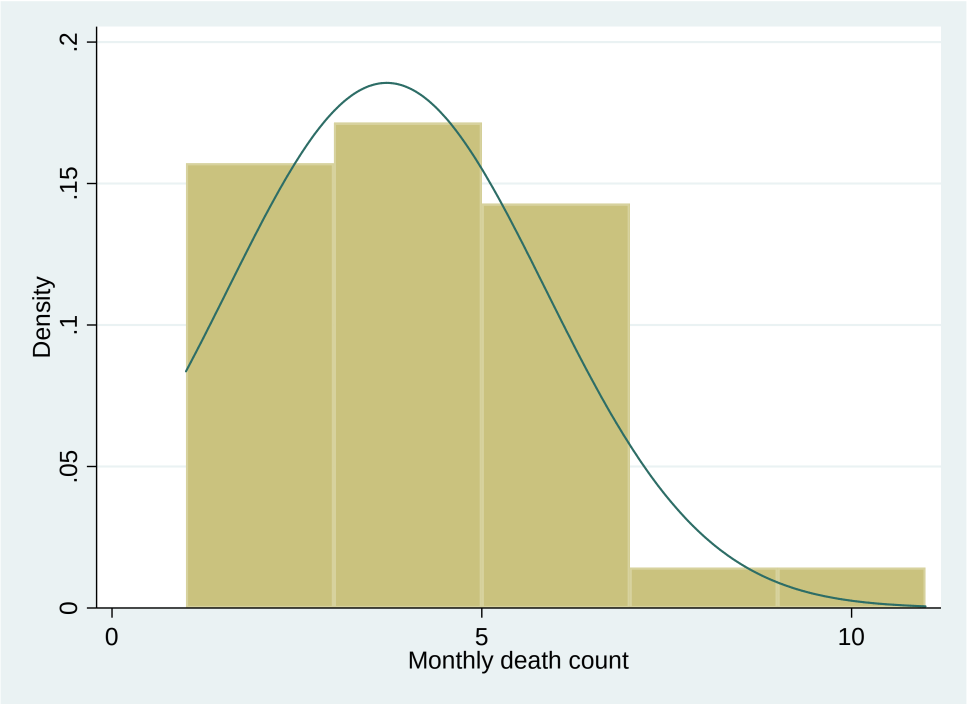

Figure S15: Assumption checking plot to perform segmented regression (Aged 20 to 39: autocorrelation).

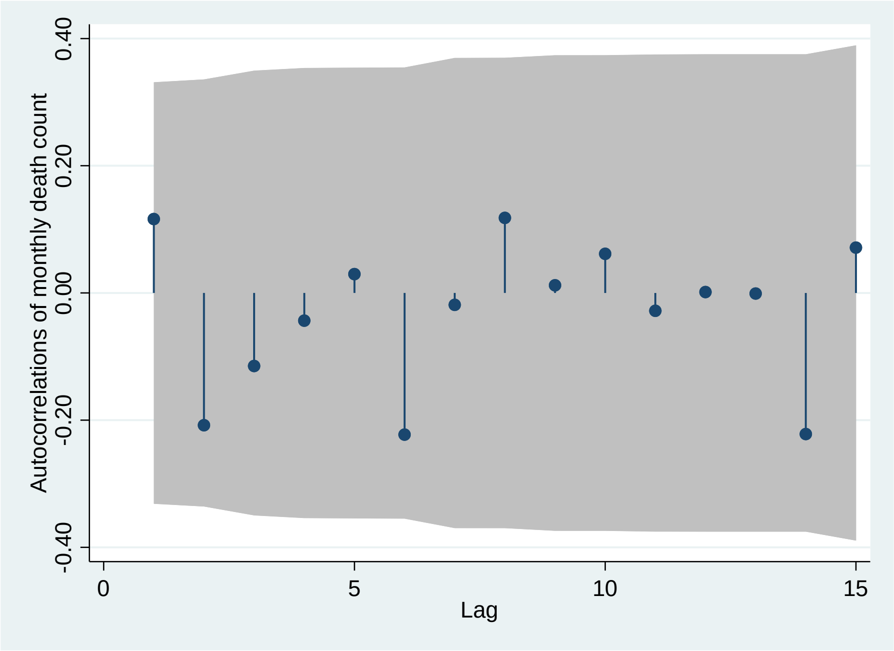

Figure S16: Assumption checking plot to perform segmented regression (Aged >= 40: normality checking).

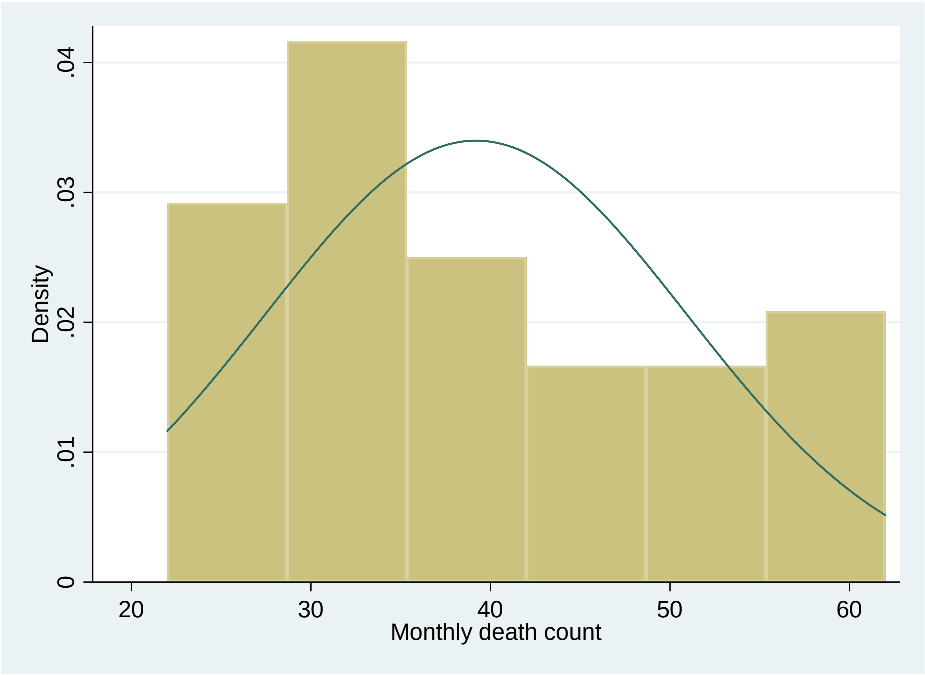

Figure S17: Assumption checking plot to perform segmented regression (Aged >= 40: autocorrelation).

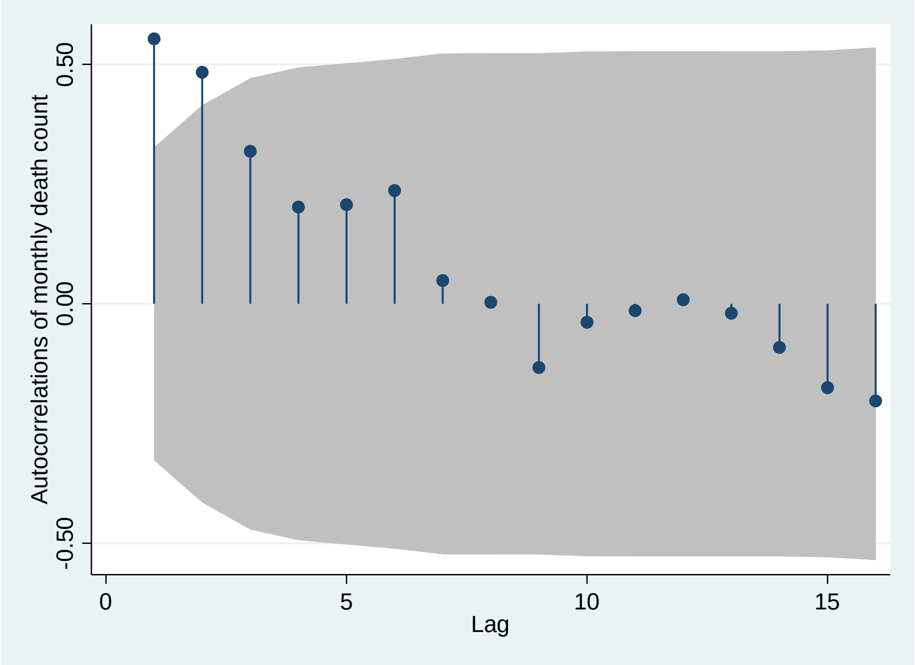

Figure S18: Assumption checking plot to perform segmented regression (Educated: normality checking).

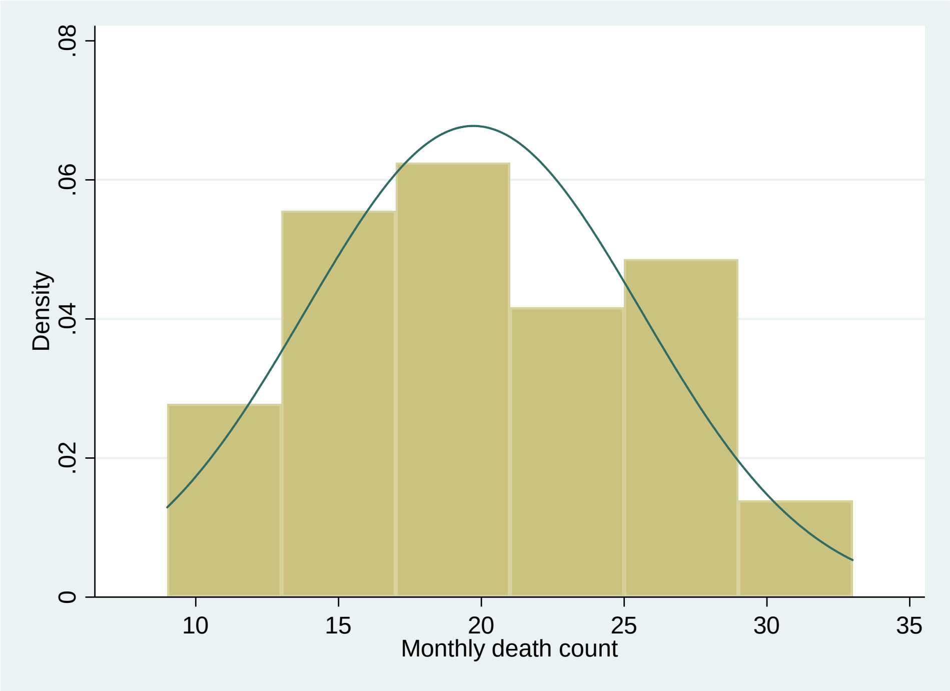

Figure S19: Assumption checking plot to perform segmented regression (Educated: autocorrelation).

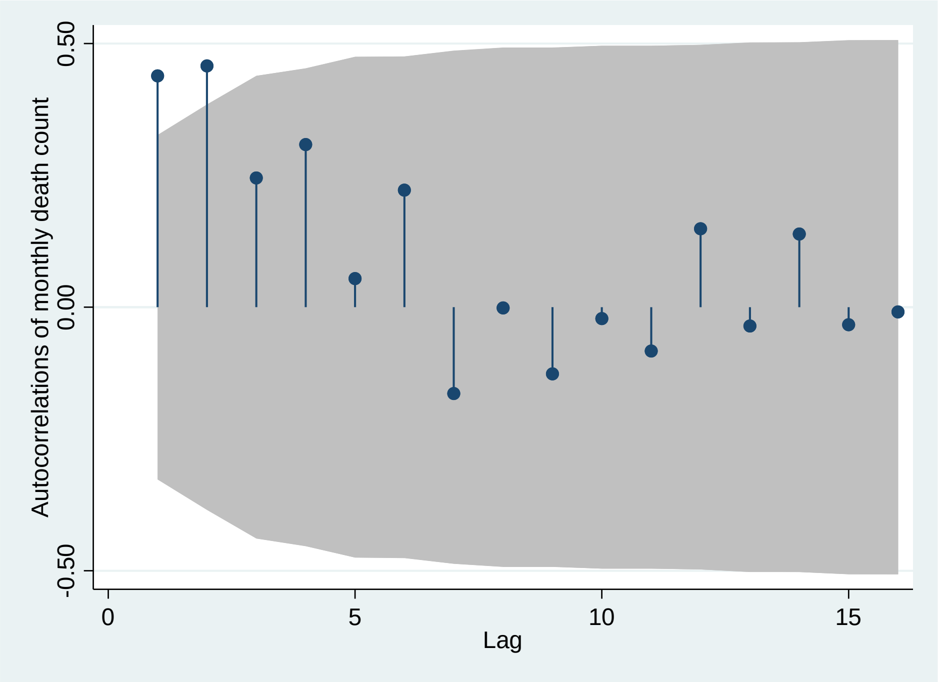

Figure S20: Assumption checking plot to perform segmented regression (No education: normality checking).

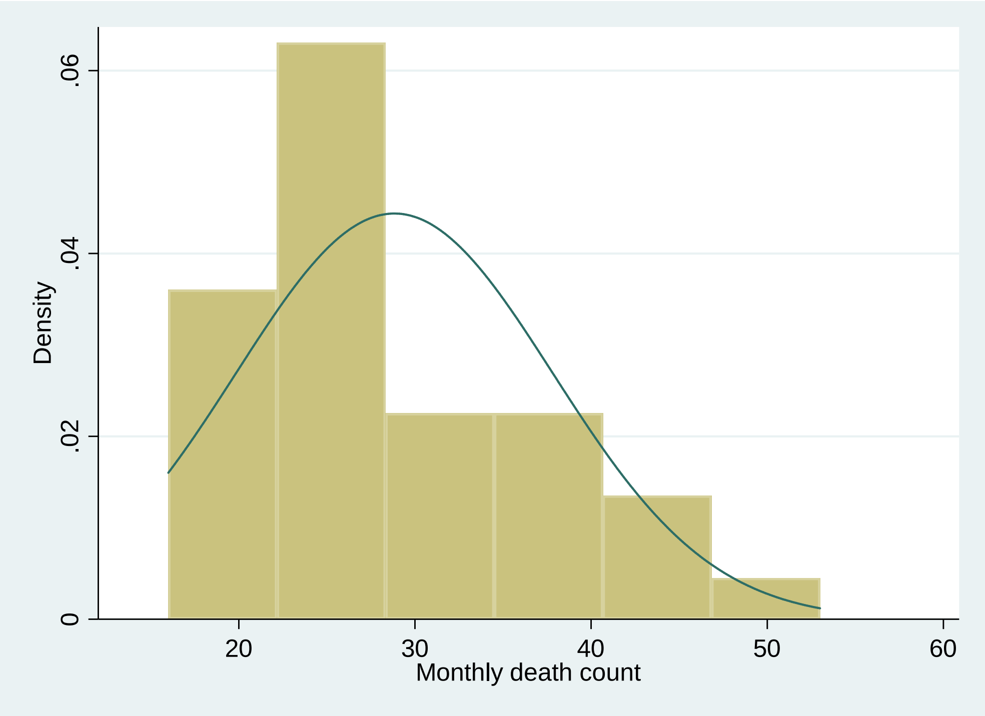

Figure S21: Assumption checking plot to perform segmented regression (No education: autocorrelation).

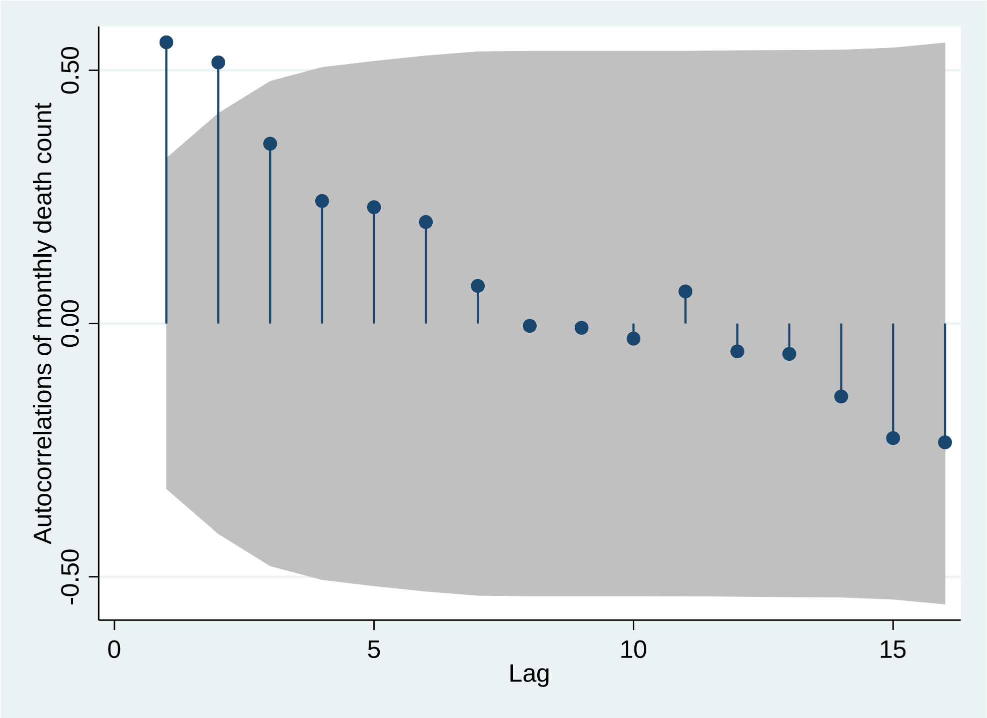

Figure S22: Assumption checking plot to perform segmented regression (Wealth-low: normality checking).

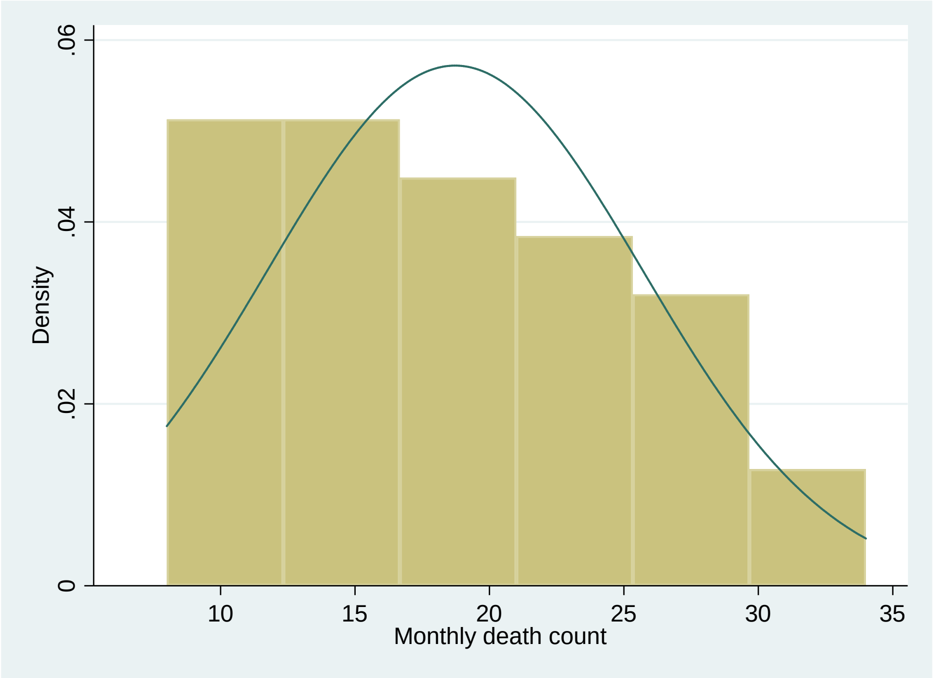

Figure S23: Assumption checking plot to perform segmented regression (Wealth-low: autocorrelation).

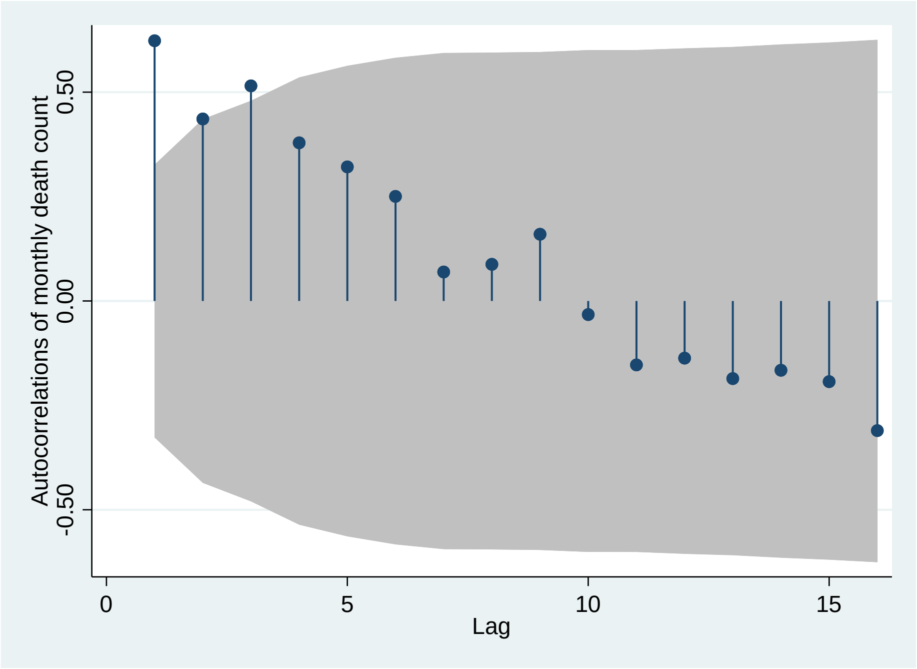

Figure S24: Assumption checking plot to perform segmented regression (Wealth-high: normality checking).

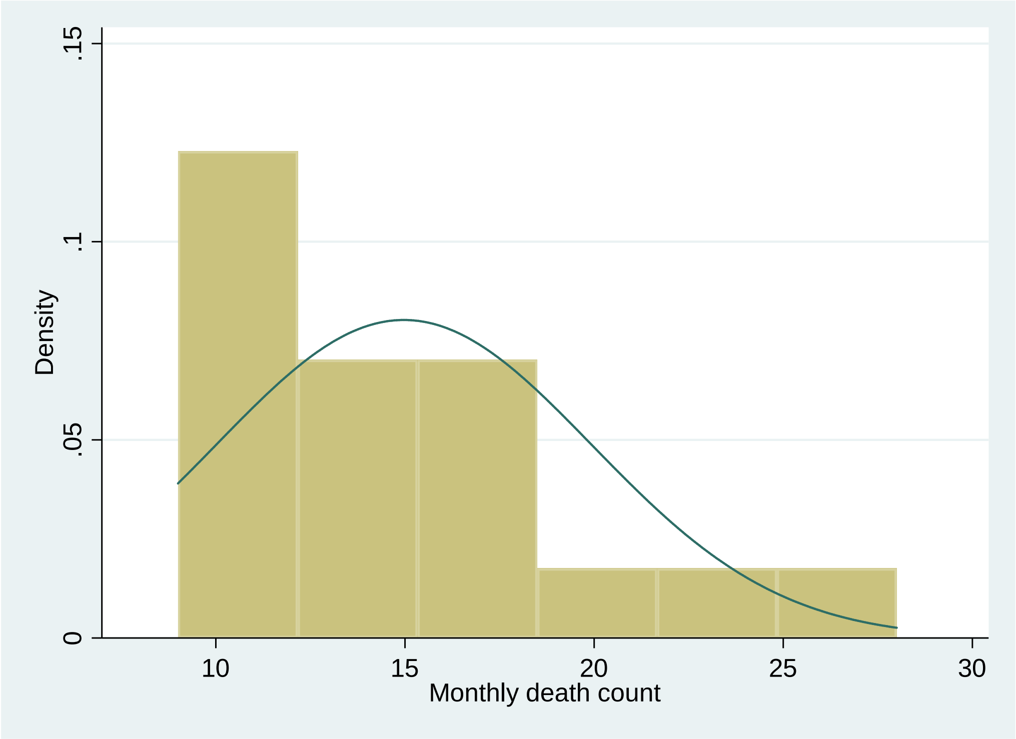

Figure S25: Assumption checking plot to perform segmented regression (Wealth-high: autocorrelation).

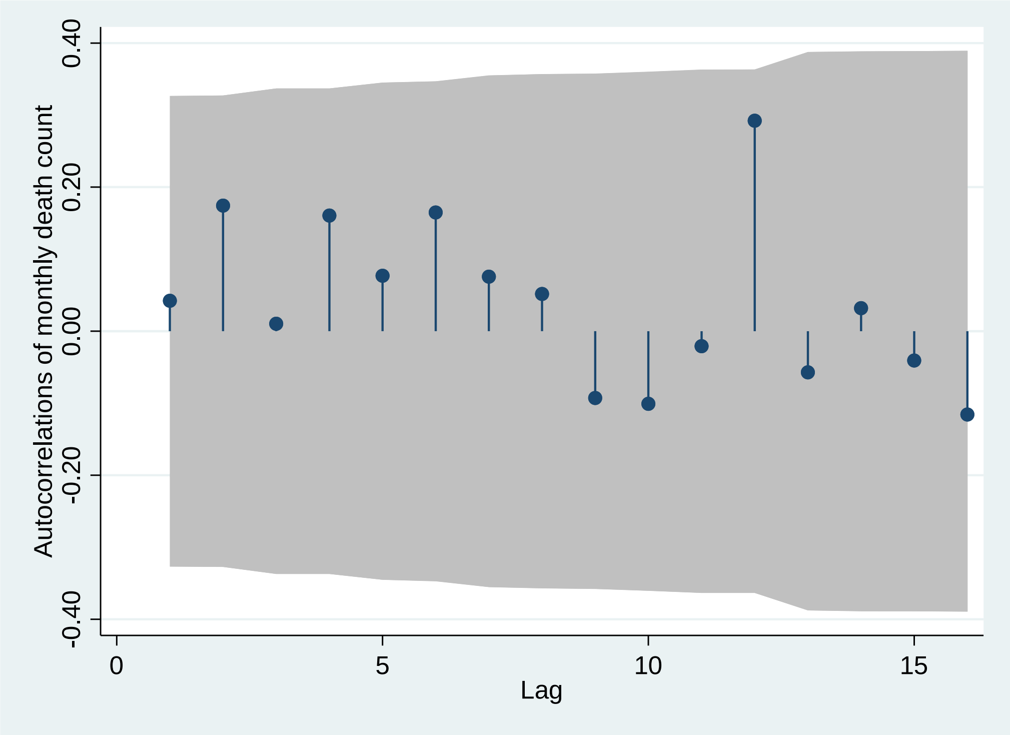

Appendix S1: Seasonality adjustment of the segmented regression model

The general form of segmented regression,

$$Y_t = \beta_0 + \beta_1 * time_t + \beta_2 intervention_t + \beta_3 * time\ after\ intervention_t + e_t$$

Where, Y_t is the mean number of deaths in month *t*; time is a continuous variable indicating time in months at time *t* from the start of the observation period; intervention (any program or event happened like COVID-19 pandemic) is an indicator for time *t* occurring before (intervention $= 0$) or after (intervention $= 1$) the covid-19 pandemic; and time after intervention is a continuous variable counting the number of months after the intervention at time t, coded 0 before the covid-19 pandemic and (1 to continue) after the covid-19 pandemic.

The seasonal pattern is an additional term in the data that repeats every 12 months [1]. According to the Fourier series expansion theorem, any repeating signal with a time period

T can be represented as a summation of sine and cosine functions. For monthly data, where

T = 12, we used Fourier series to fit the seasonality component, that is, linear combinations of sine and cosine functions. For example, to model monthly counts, the model can be written as follows [2],

$$Y_t = \beta_0 + \beta_1 * time_t + \beta_2 intervention_t + \beta_3 * time\ after\ intervention_t + \beta_s * \sin(\frac{2\pi t}{T}) + \beta_c * cos(\frac{2\pi t}{T}) + e_t$$

References:

1. Bramness JG, Walby FA, Morken G, Røislien J. Analyzing seasonal variations in suicide with Fourier Poisson time-series regression: a registry-based study from Norway, 1969–2007. *American journal of epidemiology* 2015; **182**(3): 244-54.
2. Ramanathan K, Thenmozhi M, George S, et al. Assessing seasonality variation with harmonic regression: accommodations for sharp peaks. *International journal of environmental research and public health* 2020; **17**(4): 1318.
